# Implementing gender-sensitive personalized nursing care into practice - a qualitative study with nurses from the cardiology units

**DOI:** 10.1186/s12912-026-04385-6

**Published:** 2026-02-04

**Authors:** Judith Mollenhauer, Sophia Sgraja, Ute Seeland, Martina Kloepfer, Volker E. Amelung, Clarissa Kurscheid

**Affiliations:** 1 Institute for Epidemiology, Social Medicine and Health Systems Research, Hannover Medical School, Hanover, Germany; 2 figus – Research Institute for Health- and System Design, Cologne, Germany; 3 Medical Faculty, University Hospital Magdeburg Otto von Guericke, Magdeburg, Germany; 4 Institute for Gender Health, Berlin, Germany

**Keywords:** Gender-sensitive, Evidence-based nursing, Implementation, Measures, Facilitators, Barriers, Focus group interviews, Nurses, Qualitative, Cardiology

## Abstract

**Background:**

Evidence-based gender-sensitive personalized nursing care (GSNC+) is considered in nursing expert standards and is the foundation for high-quality nursing care in Germany. However, the translation of GSNC+ into practice is still unknown. This qualitative study aims to identify implementation measures, facilitators, and barriers of GSNC + and to generate a nurse-focused definition of GSNC+. The overarching objective is to establish GSNC + as a foundational step toward personalized nursing care, focusing quality management on patient satisfaction.

**Methods:**

Nine semi-structured focus group interviews and accompanying poster brainstorming were conducted with nurses from hospitals of the cardiology section (*n* = 46 nurses; 39 females, 7 males) in Germany. Audiotaped data were fully transcribed verbatim. Besides, posters were document analyzed. Two independent coders conducted the content analysis with MAXQDA using theory-based deductive themes and generated inductive subthemes.

**Results:**

The data were clustered into five major themes and 19 subthemes organized by the Consolidated Framework for Implementation Research. Based on the results a nurse-focused definition of GSNC + was developed, and facilitators, barriers, and implementation measures were identified. From nurses’ perspective, the following aspects are needed to implement GSNC + in hospitals: Further education, adequate working and organizational structure, less time pressure in daily patient care, supporting management, media/marketing, and awareness and positive attitude from stakeholders (politicians, population/caregivers, health insurance and professional associations).

**Conclusion:**

Evidence-based content has been less included in the in-house standard operating procedures in practice and nurses implement GSNC+ mostly intuitive in patient care - quality aspects of patient care are neglected in the process. Disseminating such expert standards is not sufficient to bring about change in practice. A systematic strategy with adequate measures for quality management is needed to enhance evidence-based and high-quality GSNC+, as well as patients´ needs.

**Supplementary Information:**

The online version contains supplementary material available at 10.1186/s12912-026-04385-6

## Background

 Gender health gaps are inequalities in health care regarding sex and gender. Due to the different bodies of women and men (e.g. chromosomes, hormones, metabolism, sexual organs, fat and muscle distribution), diseases and symptoms occur with different frequency [1]. Women are particularly affected by underestimated pain, longer diagnostic delays, and access barriers than men [2]. Until the thalidomide incident in the 1950s/60s, research was predominantly conducted on male cells, tissue, and study participants, resulting in gaps of knowledge. The civil rights and women’s rights movements in the subsequent 1960s and 1970s intensified to deal with diversity in society [3].

The relevance of differentiation between women and men in care from a medical perspective was first identified in cardiology [4]. It was discovered that, e.g., myocardial infarctions can manifest differently in women than in men [5]. While men are more likely to present with left chest pain and dyspnea, women are more likely to present with nonspecific symptoms [6], such as abdominal or back pain, sweating, nausea, vomiting, or dizziness [7]. Studies have also shown that women are more likely to die after a heart attack and hospitalization (30-day lethality) in comparison to men [8, 9].

In the European population, women generally live longer than men (83,6 years vs. 78,2 years) [10]. Although women live longer, they spend more years with disabilities than men [11]. In addition to the gender inequalities arising from research history, global crises and care work are further reasons for women´s disadvantages and resulting in economic losses [12]. Besides, men experience gender health gaps when it comes to “classic women’s diseases” such as depression, breast cancer, or osteoporosis, and early mortality [13]. Currently, the debate on diversity, equity, and inclusion (DEI) is strengthening diversity in healthcare for particularly marginalized population groups (e.g. indigenous peoples, people with disabilities, or people of color) [14]. For example, black women suffer more from pain bias or are more likely to die during pregnancy and childbirth than white women [12].

Social and political debates in recent years have led to increasing attention on “sex” and “gender”. Other specialist areas pursued research interest in gender-sensitive care. Sex, gender, and factors of diversity are attributes to consider in medical and nursing care and are relevant parts of gender-sensitive patient-centered care.

Evidence-based gender-sensitive personalized nursing care (GSNC+) is considered in nursing expert standards and is a relevant component for high-quality nursing care in Germany [15, 16]. Since the 90s, expert standards are developed by expert working groups, organized by the German Network for Quality Development in Nursing Care (DNQP). In total, there exist 12 expert standards on the following topics: Decubitus prophylaxis, patient discharge management, pain management, fall prevention, continence promotion, care of chronic wounds, nutrition management, patients with dementia, oral health, skin integrity, physiological birth, and promotion of mobility [17]. They are divided into three areas: Structural quality, process quality, and outcome quality. Nurses have to consider standards and principles for safeguarding and further developing the quality of nursing care (§ 113 Social Security Code XI (SGB XI)).

Gender-sensitivity in nursing practice considers “sex” - the different biological and physiological characteristics of females, males, or intersex, and “gender” - the socially constructed characteristics of women and men [18] and further “factors of diversity”, such as age, sociocultural and socioeconomic status, mental health, physical performance, religion, ethnicity, culture, and sexual identity [19].

Overall, GSNC + is a young science, and research around GSNC+ remains insufficiently explored. There is an absence of reports of good nursing practices, the lack of definition of the attributes that characterize gender sensitivity in nursing practice, as well as the lack of knowledge about the facilitators and barriers to its implementation [20]. Nevertheless, there are approaches from culturally sensitive and individual nursing care in nursing education. Recently, the consideration of gender in care and research has become increasingly relevant, as shown in the following studies. The requirements of women and men are different and are expressed in different perceptions and outcome quality of medical care [21–24]. Evaluations in the context of GSNC+ show that female patients rated the hospital lower, needed privacy, suffered more from pain and perceived nurses´ behavior more unsatisfactory compared to male patients. The implication for practice was that women require more GSNC + to feel better during hospital stays [21]. A study about gender-specific nursing shows that women and men with multiple sclerosis wish for different treatments, for example, choosing different levels of support by nurses and communication [22]. Furthermore, research in residential care facilities has identified residents’ different care needs by gender. For example, women and men have different activity needs [23] and LGBTQ+ people require a particularly LGBTQ+-sensitive environment. Therefore, LGBTQ+-specific care homes were founded [24]. Overall, nursing is person-oriented, but there is no generally accepted definition for GSNC+. The professional ethos determines to treat people in need of care equally regardless of age, skin color, country of origin, or sexual identity [25]. Thus, people could be treated unequally because every human or patient is different and needs individual care. Currently, individual aspects of GSNC + are included in the expert standards. Examples of the standards are same-sex nursing care, same-sex room occupancy, consideration of further diversity factors, nutritional offers aligned with the culture or religion, different perceptions of pain in comparison between female and male patients, or incontinence aids adapted to the genital organs (female/male). However, gender-sensitive aspects are not particularly highlighted or emphasized in expert standards; the GSNC+ content is integrated into the descriptions of standards [17].

The following study examines how nursing professionals define GSNC + and which measures enhance or hinder the implementation in practice.

## Methods

### Context and setting

The status quo regarding the implementation of evidence-based nursing (EBN) has not yet been sufficiently investigated [26]. In Germany, the project “Gender health gaps in guideline-based inpatient cardiovascular medical and nursing care and implementation strategies to reduce the gap (HeartGap)” (funded by the German Innovation Fund of the Federal Joint Committee, the G-BA (01VSF22030)) evaluates the implementation status of gender-sensitive personalized care between 2023 and 2025 [27]. The project is divided into three study parts (systematic literature review, qualitative, and quantitative study). In preparation for the empirical part of the study, the nursing and medical guidelines regarding gender-sensitive content were analyzed. Keywords such as sex, gender, female, male, woman, and man were used for the screening in order to identify relevant text passages and evidence-based content. Content was identified in nine of the eleven relevant standards considered, particularly in the urinary continence standard. These findings were used to develop the empirical part of the study (e.g., interview questions).

### Objective, study design, and ethics

This qualitative study examines how nursing professionals define GSNC + and which measures enhance or hinder the implementation. Qualitative focus groups and content analysis were used to obtain practical and thorough information from the target group. The target group consists of nursing professionals from HeartGap´s pilot hospitals. The qualitative study was conducted in the German states North Rhine-Westphalia, Lower Saxony, and Saxony between June 2023 and May 2024. The HeartGap´s pilot hospitals were chosen from eight strata of selected attributes (number of inhabitants and grade of care). The strata are divided into two categories according to population size (large cities with more than 100,000 inhabitants, cities with up to 100,000 inhabitants) and the hospitals are divided into four categories according to level of care, defined as the number of hospital beds (teaching and research hospital with > 800 beds, large hospital with > 800 beds, medium-sized hospital with 501 to 800 beds, and small hospital with ≤ 500 beds). The selection process is described in the following: Each hospital of a strata in North Rhine-Westphalia, Lower Saxony, and Saxony was invited randomly and successively to enter the study. Hospitals of a stratum which followed the invitation first, were entered to the study as pilot hospitals. Between three and eight participants of different sex, age, and grade of education should be included in one focus group; one focus group was conducted at a pilot hospital [27].

We followed the consolidated criteria for reporting qualitative research (COREQ) guideline [28] (Appendix. COREQ checklist). Relevant national and European data protection regulations were obeyed during data collection, and participants’ anonymity and personal data were protected at all times.

### Recruitment, sampling, sample characteristics

From nine pilot hospitals of different sizes and regions, voluntary nursing care professionals participated in the qualitative study. The participants were unknown to the interviewers. Overall, 46 nurses attended the focus groups; the groups consisted between 2 and 11 participating nurses. Thirty-nine were female, 7 were male. Of the participants, 36 worked on a cardiology ward, 3 in the cardiac catheterization laboratory, 3 in a cardiac intensive care unit, and 2 in the central emergency department. Of these 44 nurses, two have also worked as trainers, and eleven have assumed managerial responsibilities. Two other interview participants were nursing directors. The professional experience of the nurses interviewed ranged from 1 to 43 years.

For each focus group, a one-hour interview was scheduled. As an incentive 50 € honorarium was paid to each participating nurse. The ethics committee approved the payment of the honorarium.

### Data collection

In preparation for the focus group interviews, an interview guideline was developed by the entire interdisciplinary HeartGap research team. The interview guideline is based on research questions and the theoretical background of implementation science [29–31]. The main objective of the interviews with nurses was to develop a definition of GSNC+ (part I) and to identify implementation measures, as well as facilitators and barriers of implementing GSNC+ (part II). Within the evaluation of the qualitative study, the focus groups were conducted by two experienced researchers: a health service researcher/health economist (JM, Master of Science - interviewer) and a public health researcher/health and legal scholar (SS, Master of Arts – co-interviewer); both are female. As a further supporting tool while interviewing, the whiteboard with prepared posters was used to visualize participants´ answers from brainstorming. The first prepared poster presented the question: “What does gender-sensitive nursing care mean to you?”, and the second prepared poster presented the question: “Which implementation measures, facilitators, and barriers do you know regarding GSNC+?” to the participants. The brainstorming with the help of the poster visualization on the topics of definition and implementation was recorded on the posters with keywords or short sentences. The same procedure was conducted at each focus group interview. All nursing participants agreed to record the interviews. The interview material comprises a 7-hour and 19-minute total interview time.

### Data analysis

Audiotaped data were fully transcribed verbatim and pseudonymized according to ethics standards and scientific rules by an external transcription bureau. Based on the transcripts and interview guideline, categories were formed by deductive and inductive approaches. The transcripts were analyzed using MAXQDA software [32]. Two researchers coded the transcript independently based on the developed coding system. Coded material and inductive codes were discussed and harmonized between the two researchers. Finally, the evaluators came to a consensus about the coding system, main categories, subcategories, and representative quotes, and coded transcripts. A qualitative content analysis according to Kuckartz is conducted based on the major and subthemes [33] and the Consolidated Framework of Implementation Research (CFIR). The CFIR supports implementation research while formative qualitative evaluation. The CFIR consists of a summary of established implementation theories and resembles a “meta-theoretical” approach. The framework consists of the following overarching topics: Innovation, outer setting, inner setting, individuals, and implementation process (Table 1) [30]. Nurses´ perspectives on implementation measures, facilitators, and barriers must be understood to develop a GSNC+ implementation approach.

The COREQ checklist was used to consider each of the items listed in the checklist during the research and reporting process [28].

**Table 1:** CFIR domains and definitions from damschroder et al. [30] regarding heartgap study reference, Mollenhauer et al. [20]

CFIR domain	Definitions
Innovation	The “thing” being implemented → GSNC+
Outer setting	The setting in which the inner setting exists → Political framework, education, research/science, professional association,.
Inner setting	The setting in which the innovation is implemented → Hospital
Individuals	The role and characteristics of individuals → Nurses´ attitudes
Implementation process	The activities and strategies used to implement the innovation → Implementation approach, measures

Additionally, document analysis was conducted by evaluating the 18 posters (two posters per interview per pilot hospital) of the focus group interviews to develop a generally accepted definition for GSNC+. Categories were formed inductively from the poster transcript, and the contents were analyzed.

## Results

The following presentation of results shows the deduced definition of interview evaluation and document analysis (part I) and implementation measures, facilitators, and barriers organized by CFIR domain (part II). The results were clustered into five major themes and 19 subthemes (Table 2).

**Table 2:** Overview of identified major themes and subthemes

Major themes	Subthemes
Innovation	• Differences in symptomatic • Diversity factors • Nursing activity • Communication and handling with inpatients
Outer setting	• Politics • Education • Health insurance • Population • Professional associations • Pharmaceutical sector
Inner setting	• Further education • Working and organizational structure • Management level • Time pressure
Individuals	• Positive attitudes • Negative attitudes • Awareness
Implementation process	• Media • Campaigns

### Part I: Nurses’ definition of GSNC+

The HeartGap study is conducted from the point of view of nurses, who care for inpatients with cardiological complaints in hospitals. Five categories resulted from the document analysis, which represent the main components of the definition: (1) differences in symptomatic, (2) diversity factors, (3) nursing activity, (4) organizational structure, and (5) communication and handling with inpatients (Table 3).

**Table 3:** Excerpt of poster brainstorming regarding categories

Category	Excerpt of poster brainstorming
Differences in symptomatic	• Different perception of pain • Different symptomatic of women/men • Women have silent infarctions
Diversity factors	• Cultures, ethnicity, feelings of shame • Religion • Age
Nursing activity	• Differences in treating patients (f/m/i) • Same-sex nursing care (e.g., during intimate hygiene) • Asking female patients about pregnancy before x-ray
Organizational structure	• Same-sex/-age room occupancy • Sex/gender of nurses in shift schedule • No differences in patients´ sex/gender regarding room occupancy in the intensive care unit
Communication and handling with inpatients	• Barriers because of language differences between the patient and nurse • Communication with trans*patients unclear • Addressing formally or informally between the patient and nurse is sometimes unclear

The following definition is divided into a general characterization, followed by parts of organization-related and patient-related GSNC+:

*Gender-sensitive personalized care (GSNC+) defines the professional nursing care provided to patients by including all sexes and genders (biological and social) as well as other diversity factors, such as age, country of origin, religious affiliation, language, physical and psychological characteristics, in nursing care practice*

*The organization-related GSNC+ includes same-sex and same-age room occupancy of inpatients or scheduling shifts with both female and male nursing staff*

*Patient-related GSNC+ refers to the nursing staff’s attention to gender-specific expressed symptoms (e.g. myocardial infarction) and pain of patients. Nurses pay attention to gender-sensitive and same-sex patient care (e.g. preparation for diagnostics and therapy such as cardiac catheterization via the groin) and care patient-centered for patients during hospitalization (e.g. in cultural/religious particularities of privacy or nutrition)*

## Part II: Nurses´ perspective on the implementation of GSNC+ organized by CFIR domain (Table 1)

### Innovation

Innovation according to CFIR [30] is defined as “the “thing” being implemented” and is in this context the GSNC+. Interviewed nurses have described and defined GSNC+ (part I). In the following section further facts about GSNC+ (subthemes: differences in symptomatic, diversity factors, nursing activity, communication and handling with inpatients) and their implementation are specified.

Predominantly, the term GSNC + is unknown among nurses. When they were asked if they had heard about GSNC + one of the participants answered: “To be honest, today for the first time“(ID 9).

The first association with the term was to care patient-centered and to care by intuition on patient´s needs, independent of the patient´s sex. Furthermore, almost all nurses stated same-sex care (particularly for intimate hygiene), different handling and communication between patient´s individuals with health care professionals, and protection of privacy are aspects of GSNC+.

In addition to gender, other diversity factors are named to be considered, such as age, language, country of origin, religious affiliation, nutritional practice, and physical and psychological characteristics.

In daily care in the hospital or palliative care, more attention can be paid to gender-sensitive care compared to emergency care (ID 8). In case of emergency, the patient´s stated preference of GSNC + can be secondary. Primarily, it is important to receive help in a medical emergency. Therefore, patients are not separated by gender in rooms of the intensive care unit:

*So for the intensive care unit, I can say that it’s also simply the division of the room, because you occupy what you can occupy. […] And you can’t move someone who is ventilated, so that you can put them together in a gender-matched way, that’s not possible. That’s why it’s sometimes difficult, we try to separate them with privacy screens, but that’s all we can do.”(ID 9)*

Usually, female and male patients are divided into different rooms in the specialty ward. Patients’ age, mobility, and physical condition are considered as far as possible when allocating specialty wards.

Shortages of staff and female-dominated nursing are causes for male patients to receive less same-sex nursing care. Besides, nurses mentioned that patients of different cultures and with different religions ask more for GSNC+.

*Well once, I had a younger patient in the emergency department, he was 35/38 and I think I thought about it afterwards, I think it was very unpleasant that I, a younger person, was also in the room. I had to shave his groin, just, I was trying to comfort him because I was, well, you’re alone in the emergency service. And I just asked “Is everything OK?” and he was like “Mhm.”, really shy and I’m really sorry about that in hindsight, but I could not do in another way. (ID 9)*

In comparison, female patients are taken less seriously than men when describing symptoms such as pain, nurses reported (ID 6).

The smallest group of gender are patients of LGBTQ+. Generally, the interviews show that nurses and hospital structures are not prepared to interact properly with transgender people. An interviewed nurse recommended:

*That we have to think about it before we get a [transgender] patient.* (ID 8)

Several hospitals have their own arrangements when a transgender person is hospitalized. These are for example the room allocation, the consideration of further gender assignments in the patient records, and the correct use of pronouns and forms of address:

*We have the nursing history form, […]- we actively ask how you want to be addressed and then we also include it in the nursing report. (ID 2)*

### Outer setting

In this context, the focus is on the implementation of GSNC + in hospitals (= inner setting). Outer setting according to CFIR is defined as “the setting in which the inner setting exists”. The dimensions of the outer setting that were identified are politics, education, health insurance, population, professional associations, and the pharmaceutical sector.

*Politics* provides the framework for the implementation of GSNC+. An important factor in implementing GSNC + in nursing practice is sufficient time dedicated to each patient. A statement made by the nurses is that politics should support the implementation of GSNC+: They especially refer to financial incentives, attractiveness (for male future nurses), modification of the nursing curriculum, working time regulations, and general relief. One measure to reduce the staff shortage is the recruitment of foreign staff under appropriate working conditions (e.g. adequate language skills, area of responsibility).

*It always has been said that the main point is politics, that is always mentioned, politics must do more. Yes, I don’t know to what extent they need to do more, but the image of nurses probably needs to change fundamentally, let’s say, the job description of a nurse is a big one. A lot of people who have already done the branch have actually quit and said “No, we’re not going to do it, we’re not going to put ourselves under any more stress. I’ll go into another job.* (ID 5)

Additionally, there should be more *education* about GSNC + for confident and respectful interaction with patients and transgender. Within the last years, the subject of culturally sensitive care has been established as part of education, in which content of different cultures and transsexuality is taught. Experienced nurses said that aspects of GSNC+ were formerly not taught at nursing school *“Not at all. So not for me.“* (ID 2). In training today, differences between female and male patients are taught, such as the different symptoms of a myocardial infarction. Another example is that textbooks contain the differences of intimate hygiene and catheter care. However, it should be emphasized that the special characteristics of women are mentioned marginally (ID 1). Experienced nurses have learned through the years of working with patients how to interact and talk to them individually. Nurses working in a diverse team foster a better understanding and improve interactions with patients of different ages, religions, cultures, migration backgrounds, or patients of the LGBTQ+ community.

*Otherwise, we always orient ourselves at what we’ve learned at school as a guide, and if there are students on the ward, […] then we talk to each other to see if there are any gender-specific changes. Whether anything new has been added. (ID 3)*

In Germany, *professional associations* develop expert standards, which nurses should use as guidelines. Nurses mentioned that contents of the standards are not well known.

*I purely believe the SOPs [*standard operating procedures*] at our hospital, they don’t make a distinction [between gender]. What it looks like in the guidelines now, I have no idea. (ID 7)*

One nurse suggests summarizing the contents of the standards in target group-oriented tables. Alternatively, generating the “One-minute wonders”^1^ would be another option to provide the content in a compact form.

*Yes, it’s always important that it’s written down somewhere, i.e. that there is a guiding principle, a guideline, something where it’s set out, that’s how we proceed. So that it is transparent for everyone.* (ID 4)

Nurses stated that institutions of the outer setting, like the *pharmaceutical sector* and *health insurance* hinder the implementation of GSNC+. In the pharmaceutical sector, in clinical studies, drug dosages were predominantly tested on men, so the dosages for women can be inadequate (ID 7). Besides, health insurance impedes the implementation of GSNC+. It is difficult for nurses to consider different genders or sexes in practice, as there is no option to enter a different gender on the health insurance card. There is only the option of indicating female or male. Transgender who are in the process of transitioning, are often classified incorrectly. The option to state the cisgender and the actual gender on the health insurance card could prevent misunderstandings. General education and raising awareness of gender differences in *the population* would help to reduce stigma.

### Inner setting

According to CFIR the inner setting is defined as “the setting in which the innovation (= GSNC+) is implemented”. Further education, working and organizational structure, management level, and time pressure are the dimensions of the inner setting that were identified.

Nurses told us that GSNC + is partially implemented concerning consideration of nutritional habits or separating female and male patients in rooms, except for the intensive care unit (ID 7). Additionally, in some hospitals, patients are separated into rooms concerning age or morbidity and when it comes to intimate care, nurses try to offer same-sex nursing care (ID 1, 2, 5). Furthermore, it was expressed that little was taught about GSNC + in the training and internal *further education* is desired in that regard.

Perhaps the instructors are also required to do more in the training, because it is also an important topic to bring this into the instructor’s curriculum. We cannot change apprenticeship training. (ID 3)

They point out that interprofessional education is important. (ID 7). Nurses prefer to impart knowledge about care standards, “One-minute wonders“, lectures or workshops, and compulsory education on diversity (ID 7)

*We have a few care standards here internally where you can always look them up. There is also this one-minute wonders that you can look at.* (ID 1)

*The working and organizational level* can also influence the intensity of implementation. Overall, implementing innovations in an organization requires time and the involvement of the staff (ID 1). In the following, measures and barriers in regard to GSNC + are cited.

Especially in nursing, more women than men work in the healthcare system (82% female nurses, 18% male nurses in Germany 2023 [34]). Therefore, male nurses are rarely available for same-sex nursing care for male patients (ID 1). A head nurse said that he did not consider whether female and male nurses work together in a shift but regarding nurses´ skills and harmony among staff (ID 2).

It was criticized that medical history forms are not sufficiently specific on differentiation of sex, gender, pathological history, or form of address (ID 2):

*In an ideal world, the medical history forms would have to be completely different, i.e. managed differently. For example, now man or woman […] Is the woman going through the menopause? Is that why she now has high blood pressure, which she never had before, or do we now say yes, you are too fat and have not exercised enough, which is why you now have high blood pressure, or is it really the menopause that has hit you? We do not ask anything like that.* (ID 7)

Moreover, there are no clear instructions or structures for the treatment and stay of transgender in hospitals (ID 4). In another participating hospital, there is a rule that transgender get single rooms to avoid conflicts between patients (ID 2). Up to now, it is not possible to add the cisgender or the selected gender in the hospital IT system. Particularly for transgender and non-binary patients this could potentially lead to misgendering and upsetting patients.

Another hospital tries to overcome language barriers by staff caring for patients who speak the same language (ID 9).

Interprofessional cooperation between nurses and physicians can improve the exchange of information on gender-sensitive topics, such as medication dosages.

*That affects a lot of people. Medicine, I would say, is just for the doctors, but in the end, we administer medication that has a completely different effect on women, and we then have the responsibility for administering these tablets. And it would be nice if doctors communicated this to us. Hey, keep an eye out on whatever different effect this drug may have on women. (ID 7)*

Nurses say that the *management level* does not promote the implementation of gender-sensitive care in hospitals to a sufficient degree. “Because you realize you just don’t get heard” (ID 7). To implement GSNC+, it is important that the ward manager or head physician acts as a role model, otherwise, the implementation will fail.

If it is, let’s say, of zero importance to some ward manager or head physician, then you will fail a bit. (ID 9)

The focus group (ID 2) reported that topics are discussed by management when problems arise in everyday working life. More and better-paid staff are needed to implement GSNC+. The management level is responsible for recruiting staff and ensuring fair payments. In addition, more male nurses are needed in the profession. One supportive measure is the more intensive recruitment of male nursing staff following an internship or voluntary social year. To make the profession more attractive, higher salaries would be helpful for recruiting staff (ID 5).

In one hospital, employees were officially informed and sensitized to it that the female and male forms of address should be used in communication.

At the beginning of the year, there was a message on the intranet about how you are now addressed, the employee, that you now say this officially. (ID 2)

*Time pressure* is one of the most frequently cited barriers of the inner setting, in addition to a lack of further education and ineligible organizational structures.

On the one hand, the lack of staff is responsible for the lack of time for further training on GSNC+ (ID 5 & 8). On the other hand, the time pressure leads to less consideration of individual patients and their wishes on how to be treated, e.g. same-sex nursing care, clarifying and gender-sensitive communication, or room occupancy to similar patients (age, mobility, morbidity).

*but I somehow don’t have time to get someone else and wait for them [for same-sex care]. I’ll do it quickly now* (ID 1)

### Individuals

Individuals according to CFIR is defined as “the role and characteristics of individuals”. Nurses´ attitudes to GSNC+ vary between positive and negative attitudes, as well as their awareness. Overall, the interviewers experienced an openness towards the topic. The nursing staff’s knowledge of the expert standards and especially specific contents of the GSNC+ seemed rather limited.

The *positive attitude* and openness are because the nursing staff have not dealt much with the topic so far and are interested in GSNC+: “and I want to deal with the topic because I also find it interesting, because as I said before: male, female, it doesn’t matter, does it? (laughing)” (ID 3). In addition, the holistic view of the patient is part of the nursing profession:

*Because we learn to look at the patient as a whole and to care for them according to their needs, and if they are a man but want to be a woman, then we take them that way because we are nurses, so I think that also plays a big role.* (ID 4)

Criticism and a rather *negative attitude* towards the implementation of GSNC+ were also expressed by some nurses. Particularly, when same-sex care should be considered or when it comes to the form of address and the personal pronoun chosen by the patient:

*We have to focus on the patient care and not on the fact that we have to focus on how to address the patient with the correct personal pronoun. That’s not on our minds at all, it’s not a topic, not at all* (ID 2)

Moreover, a nurse said that there is no need for implementation of GSNC + as long as there have been no problems with patients or for generating explicit standards for this purpose.

*Personally, I find it unnecessary. So - and if there are problems, you have to address them, there’s no question about that. But to develop standards for something like this, when it is, or should be, common sense. I personally think that’s a bit excessive, but hmm.* (ID 2)

The focus groups indicate that nursing staff need to be sensitized to aspects of gender-sensitive care (ID 6). Achieving more *awareness* and implementing further education for nurses could be beneficial (ID 7)

### Implementation process

The implementation process according to CFIR is defined as “the activities and strategies used to implement the innovation”. Research on GSNC + is still in its early stages, and this article initially focuses on defining the concept and identifying key areas of concern. As part of the focus groups and due to the new specialty, the innovation was described, and several implementation measures were identified rather than a holistic strategy being developed. Individual measures from the inner and outer settings are summarized in the sections above. In addition to the measures mentioned, the participating nurses emphasize the media, marketing, and public relations as strategies for creating awareness and supporting the implementation process among the population, stakeholders, and healthcare professionals.

In general, targeted public relations about gender sensitivity in medicine and nursing were considered supportive. Providing healthcare professionals with knowledge about GSNC+, media such as educational books, learning apps, “One-minute wonder”, information by social media, and the internet were requested. (ID 1, 3, 8, 9)

Nurses recommend initiating campaigns to raise awareness of the topic among the population and stakeholders (ID 6). Furthermore, generating a “gender certificate” for hospitals that act particularly gender-sensitive would be a landmark for patients (ID 9).

## Discussion

This qualitative study aims to identify implementation measures, facilitators, and barriers of GSNC + and to generate a nurse-focused definition of GSNC+. The overarching objective is to establish GSNC + as a foundational step toward personalized nursing care, focusing quality management on patient satisfaction.

The expert standards provide the frame for evidence-based nursing activities in practice [15]. The contents of the GSNC+ extracted from studies are included in the expert standards. The interviews show that although the existence of expert standards is widely known in practice, they are rarely implemented into practice, and the content is unknown. They tend to be used as a template for the hospital’s own SOPs. In all interviews, it was stated that the interviewed nursing staff and hospitals do not explicitly consider the implementation of GSNC + or do not include in SOPs. Currently, GSNC + is applied by the nursing staff by intuition.

**Fig. 1:**
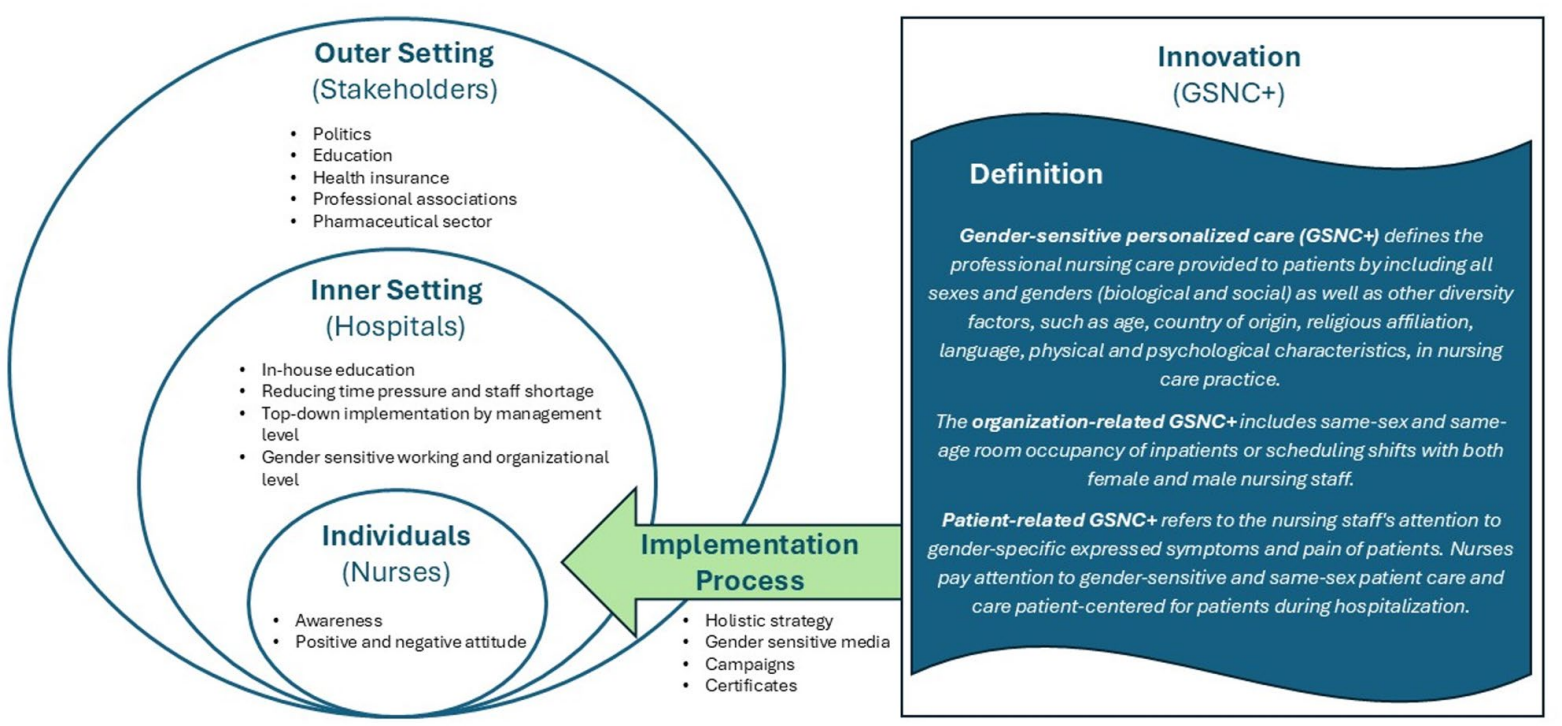
CFIR domains from Damschroder et al. with key research results

In this study, we investigated which measures are supportive of the implementation of GSNC + in the inpatient sector. In addition to the identification of the status quo of implementation and facilitators/barriers, several measures were identified at the inner setting: In-house education, gender-sensitive room occupancy, gender-mixed shift, gender-sensitive medical history forms, structures for trans*inpatients, overcoming language barriers, interprofessional cooperation, management role model, enough staff; at the outer setting: Political frame (image improvement, working hours regulations, recruitment of foreign staff), education (gender-sensitive modules in curriculum, gender-sensitive textbooks and lectures), summarized gender-sensitive information in expert standards, different options for gender classification on insurance card, gender-sensitive drug research; at the individual level: Awareness improvement; and at the implementation process: Media (gender-sensitive campaigns and gender certificate for healthcare institutions). Figure 1 shows the key research results regarding the CFIR.

Established implementation strategies can be applied to the implementation of GSNC + in hospitals. Several factors need to be considered: The prerequisites, characteristics of the innovation, and characteristics of the nurses [29, 30]. Depending on the factors mentioned, disseminating such expert standards is not sufficient to bring about change in practice. Moreover, a strategy for implementing GSNC+ requires the systematic integration of a gender perspective at all relevant levels (outer setting, inner setting, individuals, and implementation process) [30]. Büscher and Blumberg have therefore developed and tested a four-phase model for implementing expert standards with the following phases: Further education, specification and adaptation of the standard to the special requirements of the target group, compulsory introduction of the standard, and final data collection using a standardized audit instrument [15]. The four-phase model could support the development of a participative strategy for implementing GSNC + in healthcare practice successfully. In the context of implementing GSNC+, an introductory lecture on gender-sensitive content and measures from the expert standards could be held in the institutions of care (e.g., hospitals) [35]. The transfer of content to in-house processes could be discussed together with healthcare providers and subsequently tested in daily routine. Furthermore, the nurses´ attitude and adherence to follow standards are crucial influencing factors. Implementing change agents on different levels, such as management of healthcare institutions, politics, or research, could support and accompany the implementation process [20].

The guiding principle in care is that all patients should be treated equally, which is important in terms of non-discriminatory nursing [25]. Nevertheless, the guiding principle can be impedimentary in consideration of individual care tailored to each patient. Equal care for all patients is not the same as personalized care. The aim is to treat patients individually with equal quality to achieve patient-oriented outcomes [36]. Some nurses in the interview feel that explicit consideration of GSNC + or further education is not necessary, as GSNC + is part of the nursing profession. Other nursing staff perceive the population-wide discussion about gender-appropriate language and their consideration during patient communication as stressful. During the implementation process, it is important to find out whether this feeling arises from ignorance or a reluctance to embrace new ideas [29]. The integration of gender-sensitive modules in curricula or further education can support the change of attitude. The physicians´ concept of compulsory annual further education to achieve credit points could be transferred to nursing professionals. It can improve and standardize nursing care quality.

The main reasons that were mentioned in the interviews, why GSNC + is inadequately implemented were the unattractiveness of the nursing profession (especially for men), the lack of staff in hospitals, and the resulting time pressure in daily practice. This circular flow affects the lower level of tailored patient care [37–39]. Due to non-implementation of GSNC+ patients (particularly trans*patients) could experience discomfort if they do not get, same-sex-, culture-sensitive-, or individual nursing care offered [40].

Otherwise, nurses stated in the interview that they offer more GSNC+ than written in the expert standards, for instance by room occupancy. Expert standards recommend same-sex room occupancy [17]. Some hospitals consider in addition to same-sex room occupancy the patient´s age, mobility, and morbidity.

### Limitations

The developed definition and measures of GSNC + are based on focus group interviews from the inpatient sector (cardiology units). The next step should be to discuss the definition, e.g. by delphi process^2^ with nursing experts from inpatient and outpatient sectors of different specialties, to generalize the definition. Further interviews with nurses from different specialties could broaden the perspective of the GSNC+ definition.

The ethics committee approved the payment of the 50 € honorarium. However, the incentives could lead to bias in study.

## Conclusion

Evidence-based GSNC + is already considered in nursing expert standards in Germany. Disseminating such expert standards is not sufficient to bring about change in practice. A systematic strategy with adequate measures on different levels regarding innovation, outer setting, inner setting, individuals, and the implementation process is essential for successfully translating from theory to practice.

There is general agreement among politicians and stakeholders to promote gender sensitivity with regard to patient-centeredness. In the latter, as well as the current coalition agreement in Germany, the topic is part of the future political agenda. Nevertheless, gender-sensitive care has not yet reached the healthcare sector. Since currently no acute difficulties are encountered, there is no pressure to invest in GSNC + in advance for minimizing long-term consequences. Similar observations were made in regard to the implementation of prevention strategies.

The following implications can be derived for the nursing care sector in the dimensions of research and science, politics, education, and institutions of care to develop a holistic implementation strategy [20]:

- Educational level: Compulsory integration of GSNC + in the training curriculum, advanced training offers.
- Political level: Funding for GSNC+ research, mandatory framework for consideration in research.
- Research and science level: Integration of requirements to consider GSNC + in the method paper for developing expert standards, Practice-oriented instructions in expert standards.
- Institutions of care (e.g., hospitals): Integration of quality circles, internal training for GSNC+.

The focus group interviews show that GSNC + is a young science taught for a few years to future nurses. Currently, GSNC + is still applied more intuitively by nurses in practice. From the nurses´ perspective, breaking the circular flow of staff shortage, resulting in time pressure and less time to treat patients more individually, could be a first step to set the framework conditions. Further education, using media, improving awareness of nurses, and implementing change agents on different levels are supporting measures for translating the EBN of GSNC + in daily practice and to achieve the overarching aim of efficient, high-quality, personalized care.

## Supplementary Information

Below is the link to the electronic supplementary material.

Supplementary Material 1: Interview guideline (English).

Supplementary Material 2: Interview guideline (German).

Supplementary Material 3: COREQ checklist.

## Data Availability

The datasets used and/or analysed during the current study are available from the corresponding author on reasonable request.

## Footnotes

1 The “One-minute wonder” concept, which originates in England, makes use of regularly recurring waiting times during working hours. Knowledge can be imparted using focused learning charts. It should be possible to read their content in about one minute. Where possible, the ideas should come from the nursing staff - from practice for practice (https://omw.hdz-nrw.de/die-idee.html).

2 The Delphi process is a survey method in which a selected group of experts is asked to repeatedly comment on a topic. The aim is to achieve a consensus among the group. Delphi procedures are used as a formal consensus procedure [41].

## Abbreviations

DNQP: Deutsches Netzwerk für Qualitätsentwicklung in der Pflege (German Network for Quality Development in Nursing)
EBN: Evidence-based Nursing
GSNC+: Gender-sensitive nursing care +
