# Supplementary material for: Implementing gender-sensitive personalized nursing care into practice - a qualitative study with nurses from the cardiology units
Source: BMC Nurs. 2026 Feb 4;25:199. doi: 10.1186/s12912-026-04385-6 (PMC12958696; doi:10.1186/s12912-026-04385-6)
Supplement: Supplementary file 1 — Supplementary Material 1: Interview guideline (English). [file 12912_2026_4385_MOESM1_ESM.docx]

**Focus Groups – Health and Nursing Care Staff**

| **Introduction** | | | |
| --- | --- | --- | --- |
|  | | | |
| Welcome | | Welcome the participants, thank them for participating, and introduce the interviewer.  **Good morning, everyone. We are very pleased to have the opportunity to conduct this interview with you today. Thank you for participating in this focus group.**  **First, we would like to introduce ourselves: Sophia Sgraja, MHH – Institute for Epidemiology, Social Medicine and Health Systems Research; Judith Mollenhauer – figus Cologne. We are part of the operational team evaluating the project. Together with the Institute for Gender Health e.V. Berlin, we form the consortium. Medical consultation is provided by Prof. Dr. Ute Seeland (University of Magdeburg and the German Society for Gender-Specific Medicine).**  **This focus group interview is part of the HeartGap project, funded by the Innovation Fund. A focus group is a moderated discussion among a group of participants on a specific topic (in this case, gender-sensitive care). Focus groups are the best way to exchange viewpoints. As moderators, we will remain in the background, posing questions to the group, and you are invited to discuss the topic among yourselves**  **Project overview: We started the project at the beginning of the year, and it will run for two years. We are using a mixed-methods approach and have begun with a literature review and focus groups. A nationwide questionnaire survey will follow.** | |
| Objective and Procedure | | Encourage participants to engage in open conversation.  **In this interview, we would like to learn more about your experiences with gender-sensitive care in hospitals. Gender-sensitive care is already clearly represented in expert nursing standards and guidelines. However, how it is implemented in practice remains unknown. Together, we want to discuss where and why it has or has not been adopted in practice and how practical the content of the expert standards is for health and nursing care staff.**  **What measures are needed to successfully implement gender-sensitive care?** | |
| Encouragement and Data Protection | | There are no right or wrong answers—your opinions and experiences matter. Duration of the interview (approx. 60 minutes), recording.  **Please speak freely. There are no “right” or “wrong” answers. Your personal experiences and opinions are what count!**  **We would like to take about one hour for this interview.**  **With your consent, we would like to record and transcribe the interview. Your name will be replaced with a pseudonym. Only anonymized data will be reported.** | |
|  | | Start recording device | |
| Questions | | **Do you have any questions about the interview process?** | |
| Introduction Round | | **Introduction round with a poster for orientation/visualization**   - Brief introduction of the nurses (name, position, duties, how long in nursing) - Define gender-sensitive care in your own words → **collect on poster** - How do you perceive the current discourse on gender-sensitive care? - How have you perceived the development of gender-sensitive care over the past 20 years? - Do you have experience with gender-sensitive care? If so, please share—in what context? - **Opening question:** Please tell us how you would provide nursing care to a patient admitted with a heart attack who has received initial medical treatment. → When do you think gender-sensitive care is important? | |
|  | *Key questions* | | *Follow-up questions* |
|  | 1. **From Theory to Practice** | | |
|  |  | | *Transition: Where do you seek information when you need to learn about gender-sensitive care?* |
|  | Familiarity with expert standards and degree of implementation | | - Do you refer to the expert standards of the German Network for Quality Development in Nursing (DNQP)? (e.g., chronic wounds, discharge management, pain management, fall prevention, dementia, pressure ulcer prevention, promotion of urinary continence, oral nutrition, oral hygiene) - How familiar are you with the content of the expert standards, particularly regarding gender-sensitive care? - In what form would you like to receive implementation guidance for gender-sensitive care? |
|  | Education | | - What role did gender-sensitive care play in your education and further training? - Which aspects of your education addressed gender differentiation? |
|  | Implementation of Expert Standards in Hospitals | | - How do you assess the importance of this topic from upper management levels (nursing directorate/presidency)? - To what extent do your hospital’s nursing standards/guidelines include gender-sensitive aspects? - In your opinion, do they contain information on how gender-sensitive care should be implemented? - Do you perceive your hospital as open to gender-sensitive care? - What do you need (tools, manuals, evidence, etc.) to confidently implement gender-sensitive care? |
|  | 1. **Implementation in daily work** | | |
|  | Acceptance | | - How open is the health and nursing care staff in general to this topic / MHH staff in particular? - What is your personal stance on the topic? - Can you imagine implementing/intensifying gender-sensitive care in the future or engaging more with the topic? |
|  | What measures are already being used to implement gender-sensitive care? What additional measures should be introduced?  → Write on second poster | | - *What factors influence the degree of implementation of gender-sensitive care? (Information sources/training/evidence, institutional guidelines/supervisors, etc.)* - *(Outer setting: external pressure/policies/laws/local conditions and attitudes, critical incidents; Inner setting: infrastructure/information/work infrastructure/relational connection, communication, culture)* - *What characteristics or aspects related to staff or the hospital could influence gender-sensitive care in your view? (age/gender/level of care/location, etc.)* |
|  | Barriers and Facilitators | | - What factors currently hinder the implementation of gender-sensitive care in daily work? - What do you see as facilitating factors for implementing gender-sensitive care? - Where outside the hospital should efforts be made to implement gender-sensitive care? (activating keywords: education, science, public relations, …) |
|  | Communication with Patients | | - What do you think patients expect in this context? - How do you experience your communication with male and female patients (and with non-binary individuals)? - Are there specific examples where patients have demanded gender sensitivity? - When do you particularly pay attention to gender-sensitive care in patient care? (Admission, communication, direct care, discharge, interprofessional exchange…) |

|  | 1. **Conclusion and potential** |
| --- | --- |
|  | In conclusion: What are your expectations for the future? How will the topic of gender-sensitive care develop?  Is there anything else you would like to share about gender-sensitive care—something you noticed or that we haven’t discussed yet? |

- Topic interprofesionnals Where are the interfaces between gender-sensitive nursing and medicine?
