# Supplementary material for: Implementing gender-sensitive personalized nursing care into practice - a qualitative study with nurses from the cardiology units
Source: BMC Nurs. 2026 Feb 4;25:199. doi: 10.1186/s12912-026-04385-6 (PMC12958696; doi:10.1186/s12912-026-04385-6)
Supplement: Supplementary file 2 — Supplementary Material 2: Interview guideline (German). [file 12912_2026_4385_MOESM2_ESM.docx]

**Fokusgruppen Gesundheits- und Krankenpflegepersonal**

| **Einführung** | | | |
| --- | --- | --- | --- |
|  | | | |
| Begrüßung | | Begrüßung der Teilnehmer*innen, Dank für die Teilnahme, Vorstellung des Interviewers  **Guten Morgen zusammen. Wir freuen uns sehr, dass sich die Möglichkeit ergeben hat, das Interview mit Ihnen heute durchzuführen. Vielen Dank, dass Sie an dieser Fokusgruppe teilnehmen.**  **Vorab würden wir uns gerne vorstellen: Sophia Sgraja, MHH – Institut für Epidemiologie, Sozialmedizin und Gesundheitssystemforschung…; Judith Mollenhauer – figus Köln. Wir sind im Operativen Team und evaluieren das Projekt. Gemeinsam mit Institut für Gendergesundheit e.V. Berlin bilden wir das Konsortium. Medizinische Beratung Prof. Dr. Ute Seeland (Universität Magdeburg und Deutsche Gesellschaft für geschlechtsspezifische Medizin)**  **Das folgende Fokusgruppen Interview findet im Rahmen des Innovationsfonds geförderten Projektes HeartGap statt. Fokusgruppe = moderiertes Gespräch einer Gruppe von Untersuchungspersonen zu einem bestimmten Thema (hier geschlechtersensible Versorgung). Fokusgruppen sind der beste Weg, um Standpunkte auszutauschen, dabei sind wir als Moderatorinnen im Hintergrund und geben Fragen in die Runde und sie werden gebeten das Thema in der Gruppe zu diskutieren.**  **Zum Projektablauf: Wir sind Anfang des Jahres mit dem Projekt gestartet, die Projektdauer beträgt 2 Jahre. Wir führen einen Methodenmix durch und haben nun mit einem Literaturreview und den Fokusgruppen begonnen, es folgt eine deutschlandweite Fragebogenbefragung** | |
| Ziel und Ablauf | | Teilnehmer*innen zum offenen Gespräch anregen  **In diesem Interview möchten wir gerne mehr über ihre Erfahrungen mit geschlechtersensibler Pflege in Krankenhäusern erfahren. In Expertenpflegestandards und Leitlinien wird geschlechtersensible Versorgung bereits klar abgebildet. Wie es in der Praxis umgesetzt wird, ist unbekannt. Gemeinsam möchten wir diskutieren, wo und weshalb es schon in der Praxis angekommen ist oder nicht und wie praktikabel die Inhalte der Expertenstandards für das Gesundheits- und Krankenpflegepersonal ist.**  **Welche Maßnahmen bräuchte es, damit geschlechtersensible Pflege erfolgreich implementiert werden kann.** | |
| Anregung und Datenschutz | | Kein richtig oder falsch, Ihre Meinungen und Erfahrungen; Dauer des Interviews (min.), Aufnahme  **Sprechen Sie ganz frei. Es gibt weder „richtige“ noch „falsche“ Antworten. Ihre ganz persönlichen Erfahrungen und Meinungen zählen! Insgesamt möchten wir uns 1 Stunde Zeit nehmen.**  **Das Interview würden wir gerne, soweit Sie damit einverstanden sind, aufnehmen und transkribieren lassen. Ihr Name wird dabei durch ein Pseudonym ersetzt. Berichtet werden nur anonymisierte Daten.** | |
|  | | Einschalten des Aufnahmegeräts | |
| Fragen | | **Haben Sie noch Fragen zum Ablauf des Interviews?** | |
| Vorstellung/Einführung | | **Vorstellungsrunde mit Plakat als Orientierung / Visualisierung**   - Kurze Vorstellung der Pflegekräfte (Name, Position, Tätigkeiten, seit wann in der Pflege) - Geschlechtersensible Pflege mit eigenen Worten definieren.🡪 **auf Poster sammeln** - Wie nehmen Sie den aktuellen **Diskurs** zur geschlechtersensiblen Pflege wahr? - Wie haben Sie die **Entwicklung** der geschlechtersensiblen Pflege in den letzten 20 Jahren wahrgenommen? - Erfahrung mit geschlechtersensibler Pflege? - und wenn ja, berichten Sie bitte darüber/… in welchem Kontext? - **Einstiegsfrage:** Erzählen Sie uns bitte, wie ein Patient/eine Patientin, die/der mit Herzinfarkt eingeliefert wurde und die erste medizinische Versorgung erhalten hat, von Ihnen pflegerisch versorgt wird. 🡪 Wann ist es aus Ihrer Sicht wichtig geschlechtersensibel zu versorgen? | |
|  | *Leitfrage* | | *Nachfragen* |
|  | 1. **Von der Theorie zur Praxis** | | |
|  |  | | *Überleitung:* Wo informieren Sie sich, wenn Sie zum Thema geschlechtersensible Pflege Informationen benötigen? |
|  | Bekanntheit der Expertenstandards und Grad der Umsetzung | | - Orientieren Sie sich an den Expertenstandards des Deutschen Netzwerks für Qualitätsentwicklung in der Pflege (DNQP)? (Chron. Wunden, Entlassmanagement, Schmerzmanagement, Sturzprophylaxe, Demenz, Dekubitusprophylaxe, Förderung Harnkontinenz, orale Ernährung, Mundhygiene)   - Wie sehr sind Sie mit den Inhalten der Expertenstandards insbesondere in Bezug auf geschlechtersensible Versorgung vertraut? - In welcher Form wünschen Sie sich Umsetzungshinweise von geschlechtersensibler Pflege? |
|  | Ausbildung | | - Welchen Stellwert hatte das Thema geschlechtersensible Pflege in der Ausbildung und in Weiterbildungen? - Welche Inhalte in der Ausbildung hatten etwas mit der Differenzierung des Geschlechts zu tun? |
|  | Umsetzung von (Pflege-)Standards im Krankenhaus | | - Wie schätzen Sie die Bedeutung der Thematik von den oberen Führungsebenen her ein? (Pflegedirektion/Präsidium) - Inwieweit beinhalten Ihre Pflegestandards / -vorgaben des Hauses geschlechtersensible Aspekte?   - Gibt es Ihrer Meinung nach darin Informationen, wie geschlechtersensible Pflege umgesetzt werden sollte? - Erleben Sie Ihr Krankenhaus als offen für geschlechtersensible Pflege? - Was benötigen Sie (Hilfsmittel, Manuals, Evidenz etc.), um sicher geschlechtersensible Pflege umzusetzen? |
|  | 1. **Umsetzung im Arbeitsalltag** | | |
|  | Akzeptanz | | - Wie offen ist das Gesundheits- und Krankenpflegepersonal im Allgemeinen für das Thema / das MHH-Personal? - Wie stehen Sie zu dem Thema?   - Können Sie sich vorstellen, zukünftig geschlechtersensible Pflege umzusetzen/ zu intensivieren/ sich mit dem Thema zu beschäftigen? |
|  | Welche Maßnahmen werden bereits eingesetzt, um geschlechtersensible Pflege umzusetzen? Welche sollten zudem eingeführt werden?  Auf 2. Poster mitschreiben | | - *Welche Faktoren beeinflussen den Umsetzungsgrad von geschlechtersensibler Versorgung?* *(Informationsquellen/Schulungen/Evidenz, Vorgaben des Hauses/Vorgesetzten etc.) (Outer [external pressure/polities/laws/local conditions and attitudes, critical incidents] und Inner Setting [infrastructure/information/work infrastructure/ relational connection, communication, culture])* - Welche Merkmale oder Aspekte bezogen auf das Personal oder Krankenhaus könnten aus Ihrer Sicht die geschlechtersensible Versorgung beeinflussen? (Alter/Geschlecht/Versorgungsgrad/Lage etc.) |
|  | Hürden und Förderfaktoren | | - Welche Faktoren hindern derzeit die Implementierung von geschlechtersensibler Pflege im Arbeitsalltag? - Was sind aus Ihrer Sicht förderliche Faktoren für die Umsetzung von geschlechtersensibler Pflege? - Wo müsste man außerhalb des Krankenhauses ansetzen, damit geschlechtersensible Pflege umgesetzt wird? (aktivierende Schlagworte: Ausbildung, Wissenschaft, Öffentlichkeitsarbeit, …) |
|  | Kommunikation mit den Patient:innen | | - Was glauben Sie erwarten Patientinnen und Patienten in diesem Kontext? - Wie erleben Sie Ihre Pflege-Patienten Kommunikation mit weiblichen und männlichen Patienten (Wie ist die Situation bei non-binären Personen) - Gibt es konkrete Beispiele, an denen Patientinnen oder Patienten Geschlechtersensibilität eingefordert haben? - Wann achten Sie bei der Patientenversorgung (besonders) auf geschlechtersensible Pflege? (Aufnahme, Kommunikation, direkte Pflegeleistung, Entlassung, interprofessioneller Austausch…) |

|  | 1. **Ausblick und Potenzial** |
| --- | --- |
|  | Zum Abschluss: Was erwarten Sie in der Zukunft? Wie wird sich das Thema geschlechtersensible Pflege weiterentwickeln?  Gibt es noch etwas, worüber Sie zum Thema geschlechtersensible Pflege berichten möchten, was Ihnen aufgefallen ist, worüber wir noch nicht gesprochen haben? |

- Thema interprofessionelle Zusammenarbeit Wo gibt es Schnittstellen zwischen der geschlechtersensiblen Pflege und Medizin?
